# Supplementary material for: Microfluidic platform accelerates tissue processing into single cells for molecular analysis and primary culture models
Source: Nat Commun. 2021 May 17;12:2858. doi: 10.1038/s41467-021-23238-1 (PMC8128882; doi:10.1038/s41467-021-23238-1)
Supplement: Supplementary file 2 — Supplementary Information [file 41467_2021_23238_MOESM2_ESM.pdf]

Supplementary Information

Microfluidic platform accelerates tissue processing into single cells for molecular analysis and primary culture models

Jeremy A. Lombardo[1], Marzieh Aliaghaei[2], Quy H. Nguyen[3], Kai Kessenbrock[3,4], and Jered B. Haun*[1,2,4,5,6]

[1] Department of Biomedical Engineering, University of California Irvine, Irvine, CA 92697, USA.

[2] Department of Chemical and Biomolecular Engineering, University of California Irvine, Irvine, CA 92697, USA.

[3] Department of Biological Chemistry, School of Medicine, University of California, Irvine, Irvine, CA, 92697, USA.

[4] Chao Family Comprehensive Cancer Center, University of California Irvine, Irvine, CA 92697, USA.

[5] Department of Materials Science and Engineering, University of California Irvine, Irvine, CA 92697, USA.

[6] Center for Advanced Design and Manufacturing of Integrated Microfluidics, University of California, Irvine, Irvine, CA, 92697, USA

Supplementary Note 1, Evaluation of pump and device recirculation using MCF-7 cells.

We first tested the effect of repeatedly recirculating through the peristaltic pump and minced digestion device using the MCF-7 human breast cancer cell line. This is a strongly cohesive cell type that retains a significant number of aggregates after routine cell culture, and thus requires more powerful dissociation methods.[1] Prior to experiments, confluent monolayers were briefly digested with trypsin-EDTA, centrifuged, and resuspended in PBS containing 1% BSA (PBS+). Sample was then loaded into peristaltic tubing that was either looped through the pump or connected to a minced digestion device. Following recirculation for different periods of time at different flow rates, sample was collected for measurement of single cell number and viability (propidium iodide exclusion) using a Moxi flow cytometer. Results are presented in Fig. S1, with cell numbers normalized to the control. We found that recirculation through the pump alone and the minced digestion device were both associated with a modest decrease of ~10 to 20% for all conditions tested, which was significant in many cases (Figs. S1a and b). Cell viabilities were consistently ~80%, similar to control.(Figs. S1d and e). We note that it was possible for cell number to increase due to aggregate dissociation or decrease due to cell destruction, and both of these factors should increase with hydrodynamic shear stress. Since total shear varied considerably across the conditions, both in terms of flow rate and processing time, our results suggest that the small decrease in cell number observed was associated with hold-up within the system or cell loss during transfer steps.

Next we tested recirculation through the branching channel dissociation device. Previous work with this technology utilized a back-and-forth approach, which was achieved using a syringe pump.[1,2] We utilized the new integrated dissociation/filter device for this study, but flow was recirculated only through the dissociation portion and not passed through the nylon filters so as to avoid confounding the results. Cell numbers obtained after recirculating at 5, 10, and 20 mL/min for 0.5, 1, 4. and 10 min are presented in Fig. S1c. No changes were observed at the 5 mL/min flow rate. At 10 mL/min, we found that cell number increased modestly for short

recirculation times, while longer recirculation enhanced single cell recovery by up to 2.5-fold. The 20 mL/min flow rate resulted in 2 to 4-fold increases for each time point. However, cell viability dropped precipitously for the conditions that provided the largest increases in single cell number (Fig. S1f). We note that the modest increase in cell number observed at 10 mL/min for short recirculation times, on the order of ~20%, is consistent with our previous work using a syringe pump and a back-and-forth format.[1] Moreover, the correlation between very large increases in single cell number and low viability was previously seen for the filter device when very small pore sizes (5 and 10 μm) were used.[3] Based on these results, we chose 10 mL/min as the optimal flow rate for the integrated dissociation/filter device, and focused on employing shorter processing times in order to increase single cell yields without compromising cell viability. We note that 10 mL/min is also the flow rate originally used with the filtration device.[3]

Supplementary Note 2, Platform optimization using murine kidney.

The new minced digestion device and integrated dissociation/filter device were separately optimized using murine kidney samples, and results for epithelial cells are presented in the main text and Fig. 2. Single leukocytes were also quantified by flow cytometry via CD45, and results are presented in Fig. S2. From the digestion device optimization study, we found that leukocyte yield (Fig. S2a) and viability (Fig. S2b) followed similar trends as epithelial cells. Leukocytes increased with recirculation time in the digestion device, exceeding the control at 60 min, but by a more modest ~30%. Moreover, both static and interval formats produced similar results. We did find that leukocyte viability was higher with digestion device processing for all but the 60 min interval. We then investigated whether the integrated dissociation/filter device could further enhance single cell yield following 15 min of digestion device processing. For leukocytes, recovery did not change for a single pass and decreased modestly with recirculation (Fig. S2c). Relative to the 15 min control, microfluidic device processing produced 7-fold more

cells. Leukocyte viability displayed an upward trend with additional processing, but differences were not significant (Fig. S2d).

Supplementary Note 3, Single cell analysis of murine kidney.

The full microfluidic platform was evaluated using murine kidney samples, and results for epithelial cell, endothelial cell, and leukocyte numbers are presented in Fig. 3 of the main text. Single RBCs were also quantified by flow cytometry via TER119, and results are presented in Fig. S3. RBCs generally eluted at earlier timepoints for device processing, with nearly 50% recovered in the 1 min interval. A significant portion of these RBCs can likely be attributed to blood that was released during organ harvesting and mincing. However, RBCs did still increase with digestion time for controls, indicating that the digestion device may rapidly wash out cells and blood from within undigested tissue. We also assessed cell viability by flow cytometry via 7-AAD dye, and results are presented in Fig. S4. Epithelial viability was highest, at ~95% for all control and device conditions (Fig. S4a). Endothelial (Fig. S4b) and leukocyte viabilities (Fig. S4c) ranged from ~60% to 90%, with the 60 min control at ~70% for both cases. Device processing resulted in higher viabilities for endothelial cells at all conditions except the 1 min interval, and leukocytes were elevated at the 15 min time points (static and interval).

We also performed scRNA-seq on kidney samples and identified seven cell clusters that are presented and analyzed in Fig. 4 of the main text. Sequencing quality control metrics are shown in Table S2, including mean reads per cell, mean UMI, median gene, total gene, and reads confidently mapped to genome/exonic regions/transcriptome. Results were similar for the control and both device time points. The fraction of reads in cell was also presented, and the high percentages indicate that ambient RNA contamination was minimal following sorting by FACS. To confirm kidney cell cluster annotations, a cell scoring method[4] was used by implementing the “AddModuleScore" function from Seurat to compare marker gene signatures from each of the main cell clusters (Fig. S5a) and subclusters (Fig. S5b) to established

datasets.[5,6] We note that each of the seven cell clusters are represented in the control and both microfluidic processing conditions (Fig. S6a). We also evaluated the LOH, DCT, CD, & MC cluster by separating into the four different cell types. These correspond to the loop of Henle, distal convoluted tubule, collecting duct, and mesangial cells, which are each displayed in a UMAP diagram (Fig. S6b). The numbers obtained for each of these cells types are given in Fig. S6c, relative to the entire population. Each of these cell types were depleted in the 15 min platform interval, while the 60 min platform interval contained a proportional representation. We do note a slight enrichment of LOH cells and depletion of CD cells in the 60 min interval.

To facilitate correlations between scRNA-seq and flow cytometry results, we inspected for gene expression of *EpCAM*, *CD31*, and *CD45*. *EpCAM* was highly expressed predominantly in the main DCT, LOH, CD, & MC cluster (Fig. S7a), including each of the cell subsets (Fig. S7b). Proximal tubules were predominantly negative for *EpCAM*, possibly due to low basal expression and a potential secondary factor such as low protein turnover. CD45 was highly expressed in the macrophage, B lymphocyte, and T lymphocyte clusters (Fig. S7c), and *CD31* was highly expressed in the endothelial cluster (Fig. S7d), as expected. In order to make quantitative comparisons, we made two assumptions. First, we inspected the cell numbers obtained by flow cytometry in Figs. 3a-c and deduced that microfluidic processing produced approximately equal number of total cells in the 15 min interval and ~50% more cells in the 60 min interval, relative to the 60 min control. Second, we considered that all proximal tubules, as well as all DCT, LOH, CD, and MC subtypes, are EpCAM positive. Based on these assumptions, we weighted the population percentages obtained for the 60 min device interval in Fig. 4b by 1.5 and added it to the 15 min values to estimate an aggregate value for the microfluidic platform. Results are presented in Table S3, which also includes normalization to the 60 min control and calculation of aggregate population percentages. Although these estimates require caveats, they do closely match flow cytometry results in Figs. 3a-c, with ~2.5-fold more epithelial cells (proximal tubule, DCT, LOH, CD), ~2- to 2.5-fold more leukocytes

(macrophage, B and T lymphocytes), and ~4-fold more endothelial cells produced with the microfluidic platform relative to the 60 min control. Moreover, aggregate population percentages for microfluidic processing were generally comparable to the 60 min control in Fig. 4b, with the exception that endothelial cells were enriched. The relative population percentages obtained from scRNA-seq do not match flow cytometry results in Fig. 3d, however, likely due to differential sorting or droplet encapsulation of these cell types. We observed very few cells positive for the podocyte markers *Nphs1* or *Nphs2* in any of the conditions (Fig. S8), suggesting that podocytes were not isolated from kidney.

A stress response score was determined based on expression of 140 genes, as shown in the main text Fig. 4c. To provide more resolution to this result, the expression of 12 selected stress response genes are shown in Fig. S9 for each cell type. These results confirm that stress responses were generally similar between the control and device at 60 min, while values were lower for the 15 min device condition.

Supplementary Note 4, Processing and single cell analysis of murine breast tumor tissue.

We first optimized the minced digestion device and integrated dissociation/filter device separately using a murine breast tumor model (transgenic MMTV-PyMT). Samples were processed using the minced digestion device for 15, 30, or 60 min, and generated ~2- to 2.5-fold more epithelial cells than controls at the same time points (Fig. S10a). Epithelial cell viability was lower for controls than for device conditions at all digestion times (Fig. S10b). Next, we passed samples through the integrated dissociation/filter device following 15 min treatment with the digestion device. A single pass was found to be optimal in terms of epithelial cell yield (Fig. S10c) and viability (Fig. S10d), similar to kidney.

The full microfluidic platform was then evaluated, and results for epithelial cell, endothelial cell, and leukocyte numbers are presented in Fig. 5 of the main text. We also assessed cell viability by flow cytometry via 7-AAD dye, and results are presented in Fig. S11.

Epithelial cell viabilities were ~80% for all conditions except the 60 min control and 15 min device interval, which decreased to ~70% (Fig. S11a). Endothelial cell viability was generally low at ~60% (Fig. S11b). However, the 1 min device interval was higher at 75%, while the 60 min control and 15 min device interval were lower at 50% and 40%, respectively. Leukocyte viability remained ~80% for all but the 60 min control, which was ~60% (Fig. S11c).

We also performed scRNA-seq, and identified six cell clusters that are presented and analyzed in Fig. 6 of the main text. Sequencing quality control metrics are also shown in Table S2, and were similar for the control and both device time points. Epithelial cells were the predominant cluster, and we further identified three sub-clusters that corresponded to luminal, basal, and proliferating luminal cells (Fig. S12a). These sub-clusters were associated with expression of *Krt14*, *Krt18*, and *Mki67* genes (Fig. S12b). Population percentages, relative to the full population, are presented in Fig. S12c. The luminal subtype was enriched in the 15 min interval, the basal subtype was enriched in the 60 min interval, and the proliferating luminal was under-represented at both time points.

We correlated scRNA-seq results to flow cytometry in a similar manner as kidney. *EpCAM* was now well-correlated with the main epithelial cluster (Fig. S13a), as well as each sub-cluster (Fig. S13b). *CD45* was highly expressed in macrophage, T lymphocyte, and granulocyte clusters (Fig. S13c), while *CD31* was highly expressed in the endothelial cluster (Fig. S13d). Microfluidic platform results were then aggregated using the same approach described for kidney, with 60 min interval results weighted by 1.5 and added to 15 min interval values, and results are presented in Table S5. These estimates again matched flow cytometry results for each cell population (Figs. 5a-c), with ~2-fold more epithelial cells and ~4-fold more endothelial cells produced with the microfluidic device relative to the 60 min control. Leukocyte values relative to the control were 3-fold higher for macrophages, 2.5-fold higher for T lymphocytes, and 20% lower for granulocytes. Notably, we found substantial increases for fibroblasts (>10-fold) and basal epithelial cells (>6-fold) with microfluidic processing. Aggregate

population percentages for the microfluidic platform were generally comparable to the 60 min control in Fig. 6b, but with significant enrichment of macrophages, endothelial cells, and fibroblasts. As with kidney, the relative population percentages obtained from scRNA-seq do not match flow cytometry results in Fig. 5d, again suggesting differential sorting or droplet encapsulation for different cell types.

Lastly, the expression of 12 selected stress response genes are shown in Fig. S14 for each cell type. These results confirm that stress responses were generally similar across the control and device conditions.

Supplementary Note 5, Isolation of hepatocytes from murine liver.

We tested the minced digestion device and integrated dissociation/filter device separately using murine liver, and found that the integrated device decreased hepatocyte yield (Fig. 7a) and viability (Fig. 7b). We hypothesized the second filter, with a pore size of 15 μm, was too small for large and fragile hepatocytes. Therefore, we created a modified version of the integrated dissociation/filter device that omitted the second filter. After processing liver for 15 min with the minced digestion device, the cell suspension was passed through the modified dissociation/filter device one time, which increased hepatocytes by 30% relative to the digestion device alone and by nearly 3-fold relative to the control (Fig. S15a). Hepatocyte viability was preserved, remaining >85% for all conditions (Fig. S15b).

The full microfluidic platform (with modified filter device) was then evaluated, and results for hepatocyte, endothelial cell, and leukocyte numbers are presented in Fig. 7 of the main text. We also assessed cell viability by flow cytometry via 7-AAD dye, and results are presented in Fig. S16. Hepatocyte viability remained at ~90% for most conditions tested (Fig. S16a). A small increase was observed for static or interval processing conditions, but values were not significantly different than controls. Endothelial cell (Fig. S16b) and leukocyte (Fig. S16c) viabilities followed similar trends seen in hepatocytes, and were between ~70% and 85%.

Supplementary Note 6, Isolation of cardiomyocytes from murine heart.

We tested the minced digestion device, with and without the integrated dissociation/filter device using murine heart. This included both the original integrated device and the modified version without the 15 μm filter that was created for liver. We found that after processing heart tissue for 15 min, cardiomyocyte numbers and viability were unchanged for each case (Fig. S17). As a result, we selected to use the standard version of the integrated dissociation/filter device with both 50 and 15 μm filters for heart tissue.

The full microfluidic platform was then evaluated, and results for cardiomyocyte, endothelial cell, and leukocyte numbers are presented in Fig. 8 of the main text. We also assessed cell viability by flow cytometry via Zombie Violet dye, and results are presented in Fig. S15. Cardiomyocyte viability was ~70% for controls, while values for device processing were all at ~75-85% (Fig. S18a). Viabilities for endothelial cells (Fig. S18b) and leukocytes (Fig. S18c) were >80% for all device and control conditions.

Supplementary Table 1. Coefficient of variation values for kidney samples at different processing conditions.

Condition	Epithelial Cells	Endothelial Cells	Leukocytes
Control 15 m	24.5	15.4	10.4
Control 60 m	20.2	13.2	17.3
Static 15 m	27.2	25.3	20.3
Static 60 m	17.1	19.2	11.1
Interval 1 m	25.4	18.5	11.3
Interval 15 m	7.5	11.7	10.2
Interval 60 m	7.8	6.5	1.2

Supplementary Table 2. scRNA-seq metrics for kidney and breast tumor samples.

Cond.	Tissue	Mean Reads/ Cell	Mean UMI	Median Gene	Total Gene	Reads Mapped Confidently to			Fraction Reads in Cell
						Genome	Exonic Regions	Transcript ome	
60 Control	Kidney	61626	6091	1818	20138	93.4%	78.00%	75.1%	80.6%
15 Platform		57453	8244	2076	19761	93.6%	78.5%	75.70%	91.4%
60 Platform		59596	5575	1770	21487	93.0%	74.7%	71.60%	80.9%
60 Control	Breast Tumor	44440	8412	2335	20908	90.5%	68.5%	65.5%	92.7%
15 Platform		41371	10286	2836	20895	90.4%	68.3%	65.0%	94.2%
60 Platform		47196	10788	2677	21357	91.1%	69.8%	66.8%	95.5%

Supplementary Table 3. Weighted population values for each cluster and sub-cluster in murine kidney. Population percentages for microfluidic processing in Fig. 4b were weighted (1x for 15 min and 1.5x for 60 min) and added to create total aggregate microfluidic platform values. These were normalized by the control and used to calculate total aggregate population distributions.

Cluster	Device 60 m (weighted)	Device Total (weighted)	Device Total (Norm. to control)	Device Total (%)
Proximal Tubule (S2-S3)	16.2	68.3	2.7	27.3
Proximal Tubule (S1)	27.8	61.6	2.3	24.6
Endothelial	38.9	43.7	4.2	17.5
Macrophage	32.4	34.7	1.9	13.9
LOH, DCT, CD, & MC	15.3	17.4	2.0	7.0
LOH	7.0	7.9	1.8	3.1
DCT	5.4	6.2	2.8	2.5
CD	2.1	2.3	1.8	0.9
MC	0.9	1.3	1.4	0.5
B Lymphocyte	9.8	12.7	2.3	5.1
T Lymphocyte	9.8	11.8	2.9	4.7

Supplementary Table 4. Coefficient of variation values for breast tumor samples at different processing conditions.

Condition	Epithelial Cells	Endothelial Cells	Leukocytes
Control 15 m	25.4	26.4	41.0
Control 60 m	19.9	13.1	19.5
Static 15 m	23.5	23.7	24.6
Static 60 m	12.6	9.7	27.9
Interval 1 m	14.2	37.2	25.9
Interval 15 m	14.0	17.0	11.7
Interval 60 m	12.2	21.6	14.3

Supplementary Table 5. Weighted population values for each cluster and sub-cluster in murine breast tumor. Population percentages for microfluidic processing in Fig. 6b were weighted (1x for 15 min and 1.5x for 60 min) and added to create total aggregate microfluidic platform values. These were normalized by the control and used to calculate total aggregate population distributions.

Cluster	Device 60 m (weighted)	Device Total (weighted)	Device Total (Norm. to control)	Device Total (%)
Epithelial	65.4	133.9	2.2	53.5
Luminal	59.1	125.9	2.1	50.4
Basal	4.5	5.3	6.6	2.1
Lum. Prolif.	1.8	2.7	1.2	1.1
Macrophage	43.2	61.7	3.1	24.7
Endothelial	20.4	25.0	4.2	10.0
T Lymphocyte	9.5	13.8	2.4	5.5
Fibroblast	9.8	11.6	10.5	4.6
Granulocyte	2.0	4.2	0.8	1.7

Supplementary Table 6. Coefficient of variation values for liver samples at different processing conditions.

Condition	Hepatocytes	Endothelial Cells	Leukocytes
Control 15 m	27.6	5.5	22.7
Control 60 m	26.7	8.6	15.8
Static 5 m	13.8	11.5	16.7
Static 15 m	26.5	11.7	8.6
Interval 1 m	20.6	11.3	3.7
Interval 5 m	14.2	8.4	7.7
Interval 15 m	14.5	11.5	4.9

Supplementary Table 7. Coefficient of variation values for heart samples at different processing conditions.

Condition	Cardiomyocytes	Endothelial Cells	Leukocytes
Control 15 m	13.3	35.7	26.1
Control 60 m	14.1	26.8	54.5
Static 5 m	15.7	22.8	33.6
Static 15 m	36.2	30.8	40.2
Interval 1 m	28.9	27.4	28.4
Interval 5 m	12.7	21.5	40.1
Interval 15 m	7.1	22.5	34.5

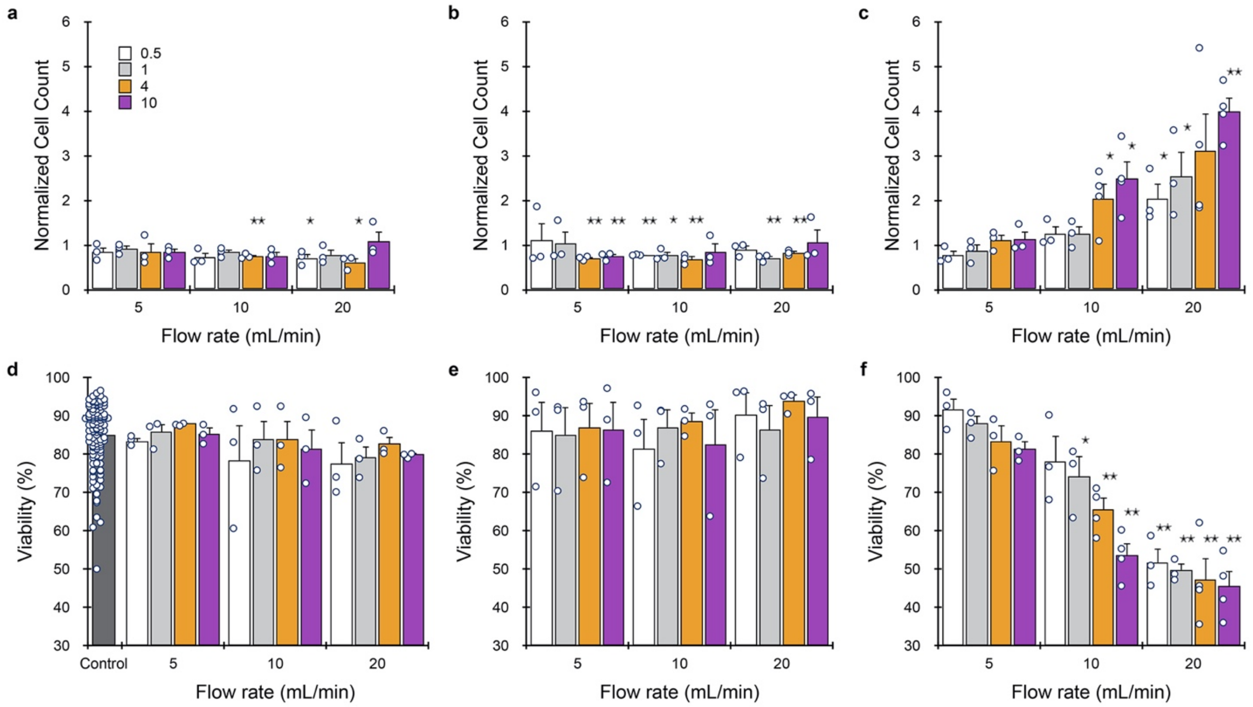

Supplementary Figure 1. Recirculation studies with MCF-7 cell line. MCF-7 breast cancer cells (n=3 or 4 independent samples for experimental conditions, n=112 independent samples for control) were continuously pumped through the (a,d) peristaltic pump, (b,e) minced digestion device, or (c,f) dissociation/filter device at different flow rates and for different time periods. (a-c) Cell counts were obtained and normalized to the control. Cell numbers decreased modestly for (a) pump alone and (b) digestion device under all conditions. (c) The dissociation device increased cell recovery for the longer time points at 10 mL/min and all time points at 20 mL/min. (d-f) Cell viability remained high for (d) pump only, (e) digestion device, and (f) dissociation device at 5 mL/min. However, higher flow rates decreased viability for the dissociation device, in a manner that correlated inversely with increases in single cell yield. Data are presented as mean values +/- SEM from at least three independent experiments. Circles indicate values for experimental replicates. Two-sided T test was used for statistical testing. Stars indicate $p < 0.05$ and double stars indicate $p < 0.01$ relative to the control. p-values for all comparisons are presented in the Source Data file.

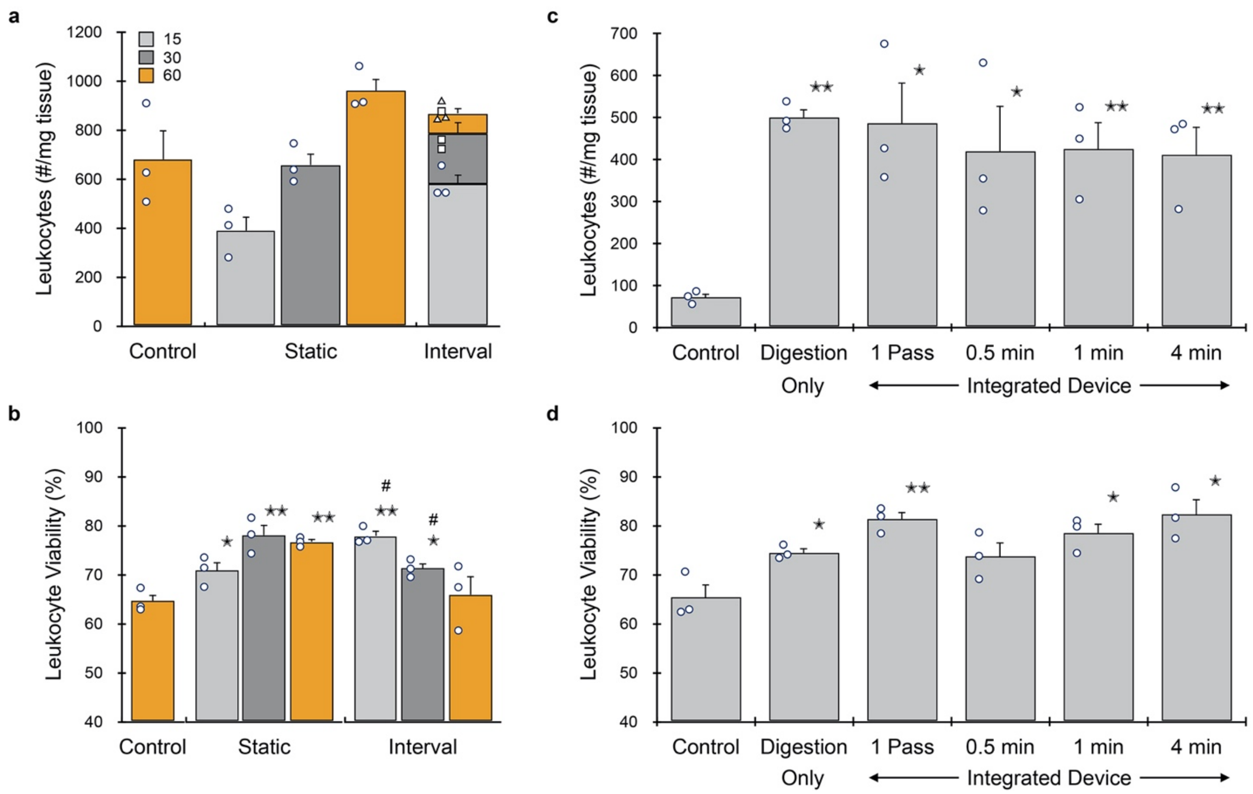

Supplementary Figure 2. Leukocyte results from optimization studies using murine kidney (n=3 independent samples). (a,b) Minced digestion device optimization under static and interval formats, compared to control that was digested for 60 min. (a) Leukocyte yield increased with digestion device processing time to ~1000/mg, exceeding the control by ~30%. Interval recovery did not affect results. (b) Viability increased from ~65% for control to >70% for all device conditions. (c,d) Integrated dissociation/filter device optimization using sample that was processed for 15 min in the digestion device, compared to control digested for 15 min. (c) Leukocyte recovery remained the same after a single pass and decreased modestly with recirculation. (d) Leukocyte viability was ~85-90% for all conditions. Data are presented as mean values +/- SEM from at least three independent experiments. Circles indicate values for experimental replicates. For the stacked plot, experimental replicates are indicated by circles at 15 min, squares at 30 min, and triangles at 60 min. Two-sided T test was used for statistical testing. Stars indicate $p < 0.05$ and double stars indicate $p < 0.01$ relative to the control. Cross-

hatches indicate $p < 0.05$ relative to the static condition at the same digestion time. p-values for all comparisons are presented in the Source Data file.

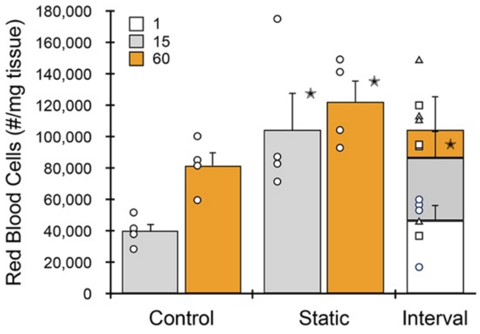

Supplementary Figure 3. Red blood cell results for murine kidney (n=4 independent samples). Most RBCs were eluted at early timepoints for device processing. Due to the high recovery after only 1 min, this time point was added to interval studies for all tissues. Data are presented as mean values +/- SEM from at least three independent experiments. Circles indicate values for experimental replicates. For the stacked plot, experimental replicates are indicated by circles at 15 min, squares at 30 min, and triangles at 60 min. Two-sided T test was used for statistical testing. Stars indicate $p < 0.05$ relative to the control at the same digestion time. p-values for all comparisons are presented in the Source Data file.

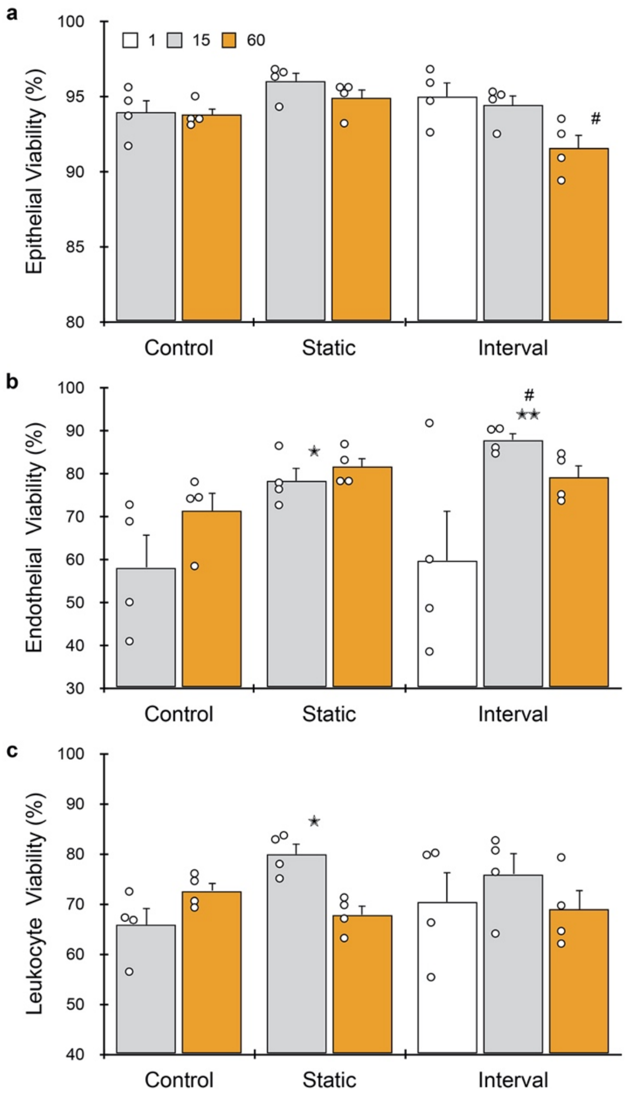

Supplementary Figure 4. Cell viability from final microfluidic platform studies using murine kidney (n=4 independent samples). (a) Epithelial cell viability was ~95% for all conditions. (b) Endothelial cell and (c) leukocyte viabilities ranged from ~60% to 90%, with the 60 min control at ~70% in both cases. Device platform processing resulted in higher viabilities for endothelial cells at all conditions except the 1 min interval, and leukocytes were elevated at the 15 min time points (static and interval). Data are presented as mean values +/- SEM from at least three independent experiments. Circles indicate values for experimental replicates. Two-sided T test was used for statistical testing. Stars indicate $p < 0.05$ and double stars indicate $p < 0.01$ relative to the control at the same digestion time. Cross-hatches indicate $p < 0.05$ relative to the

static condition at the same digestion time. p-values for all comparisons are presented in the Source Data file.

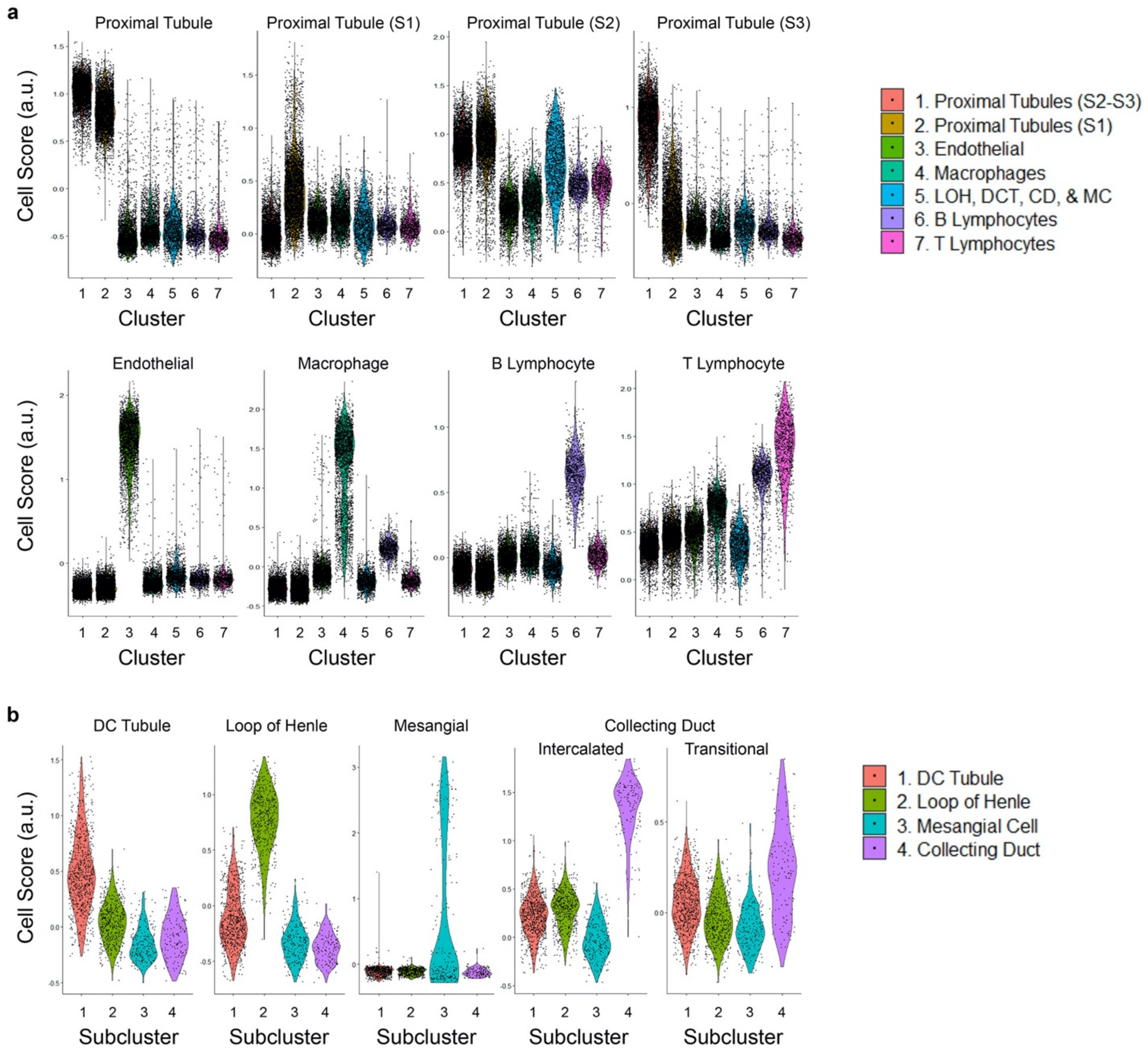

Supplementary Figure 5. Gene scoring of kidney cell types. Cell scoring results that were used to compare marker gene signatures for each of the (a) seven main clusters and (b) sub-clusters.

a

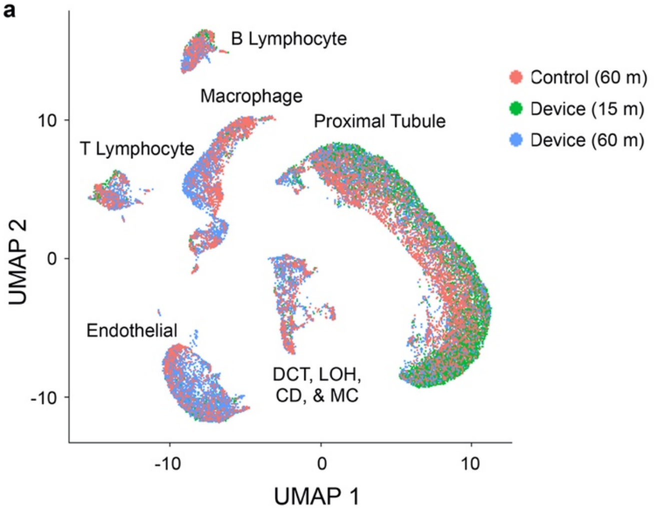

b

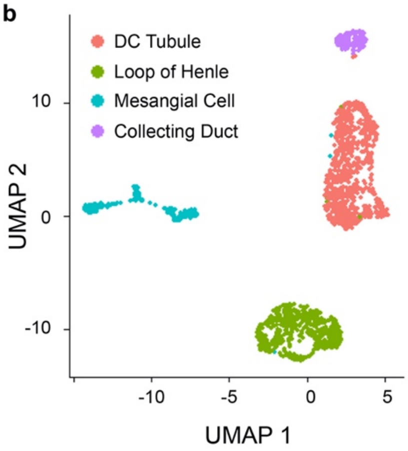

c

Cell Type	Control 60 m	Device 15 m	Device 60 m
Distal Convoluted Tubule	4.3	0.8	4.7
Loop of Henle	2.2	0.8	3.6
Mesangial Cell	1.3	0.2	1.4
Collecting Duct	0.9	0.4	0.6

Supplementary Figure 6. scRNA-seq analysis for murine kidney (n=1). (a) All seven cell clusters were represented in control and device conditions. (b) UMAP representation showing the 4 sub-cluster within the DCT, LOH, CD, & MC cluster. (c) Distributions obtained for each sub-cluster, relative to the full population.

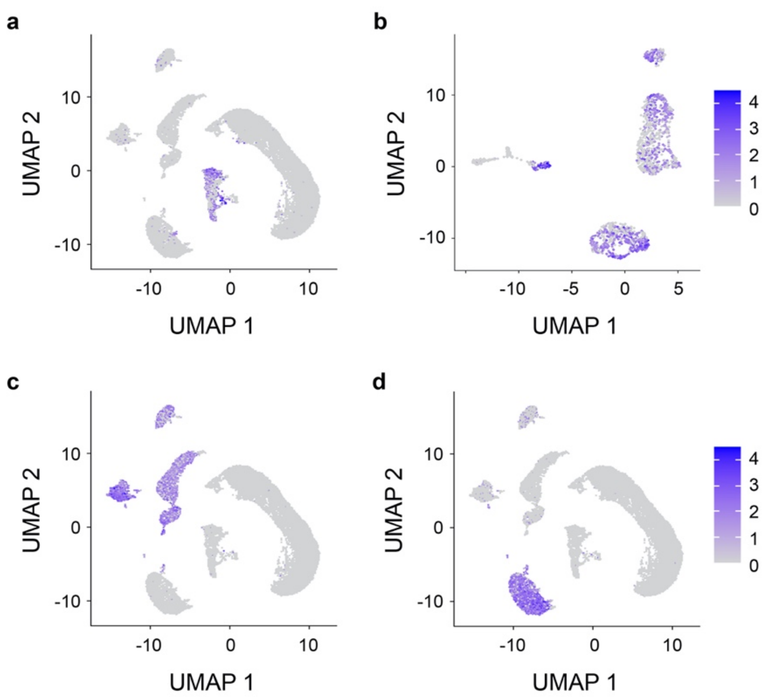

Supplementary Figure 7. Expression of *EpCAM*, *CD45*, and *CD31* in kidney clusters. (a,b) *EpCAM* was highly expressed within the (a) DCT, LOH, CD, & MC cluster and (b) each individual sub-cluster. (c) *CD45* was highly expressed in the macrophage, B lymphocyte, and T lymphocyte clusters. (d) *CD31* was highly expressed in the endothelial cluster.

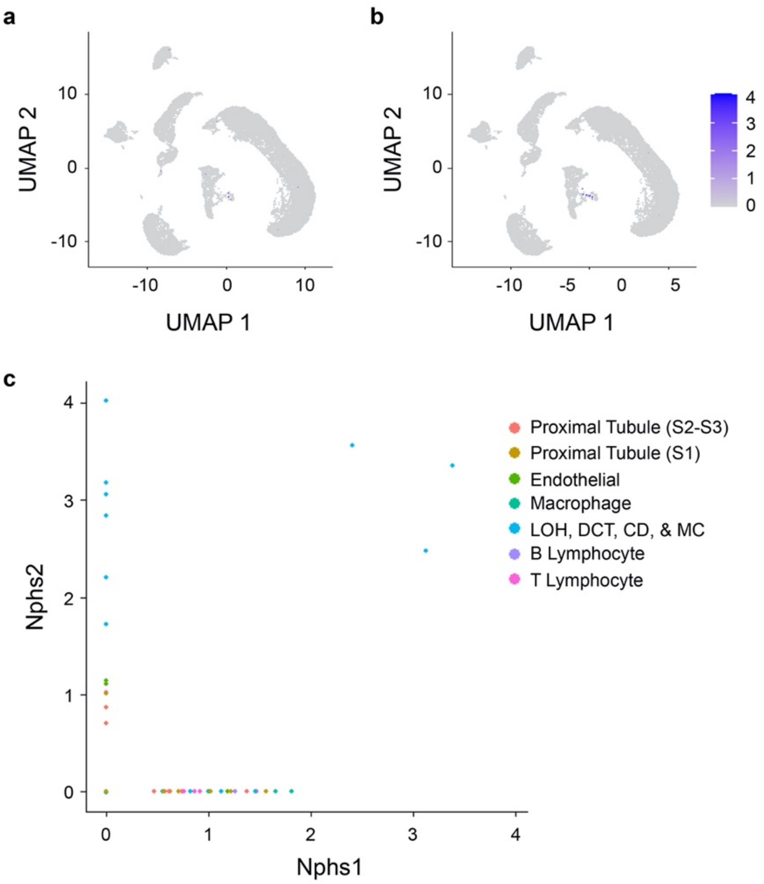

Supplementary Figure 8. Podocyte markers. Gene expression of (a) *Nphs1* and (b) *Nphs2*, with positive expression showing in only a small number of cells that were predominantly in the LOH, DCT, CD, & MC cluster. (c) Few cells were positive for both *Nphs1* and *Nphs2*.

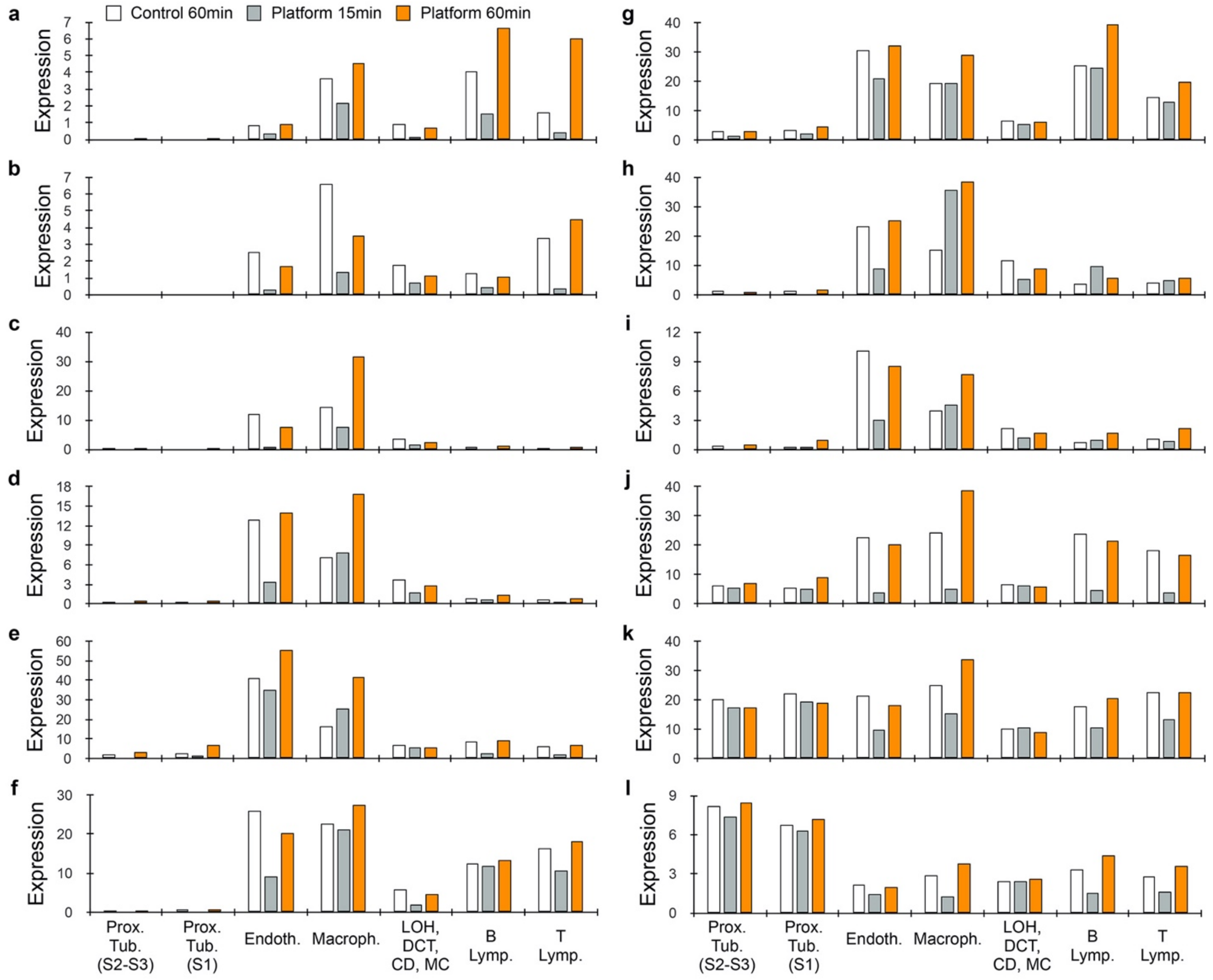

Supplementary Figure 9. Expression of select stress response genes for each kidney cell cluster. Average gene expression for common stress response genes including (a) *Nr4a1*, (b) *Gadd45b*, (c) *Atf3*, (d) *Egr1*, (e) *Jun*, (f) *Junb*, (g) *Jund*, (h) *Fos*, (i) *Fosb*, (j) *Hsp90aa1*, (k) *Hspa8*, and (l) *Hspd1*.

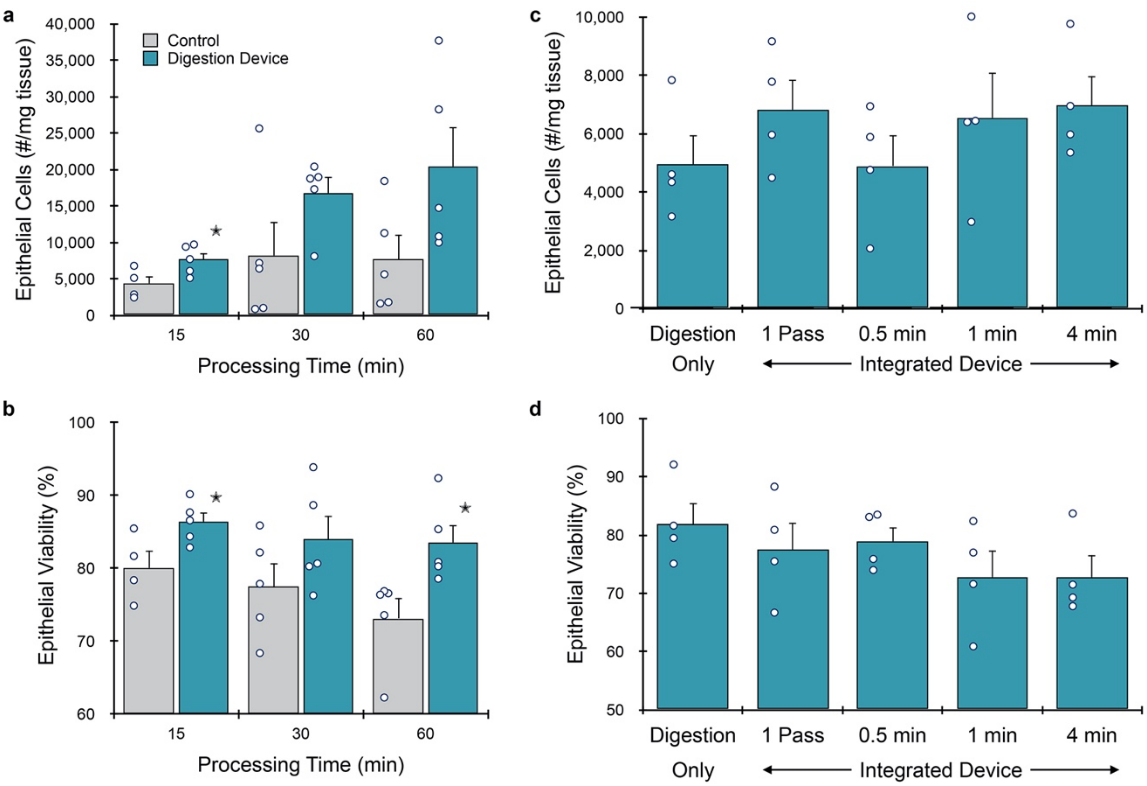

Supplementary Figure 10. Device optimization studies using murine breast tumor (n=4 or 5 independent samples). (a,b) Minced digestion device operated for different time points. (a) Epithelial cell yield increased by ~2- to 2.5-fold using the digestion device. (b) Viability was ~80% for the 15 min control and decreased slightly with time, while all device conditions were >85%. (c,d) Integrated dissociation/filter device optimization using sample that was processed for 15 min in the digestion device. (c) Epithelial recovery increased by 30% after a single pass, while recirculation produced similar or lower numbers. (d) Viability decreased slightly after dissociation/filter treatment, but changes were not significant. Data are presented as mean values +/- SEM from at least three independent experiments. Circles indicate values for individual replicates. Two-sided T test was used for statistical testing. Stars indicate $p < 0.05$ relative to the control at the same digestion time. p-values for all comparisons are presented in the Source Data file.

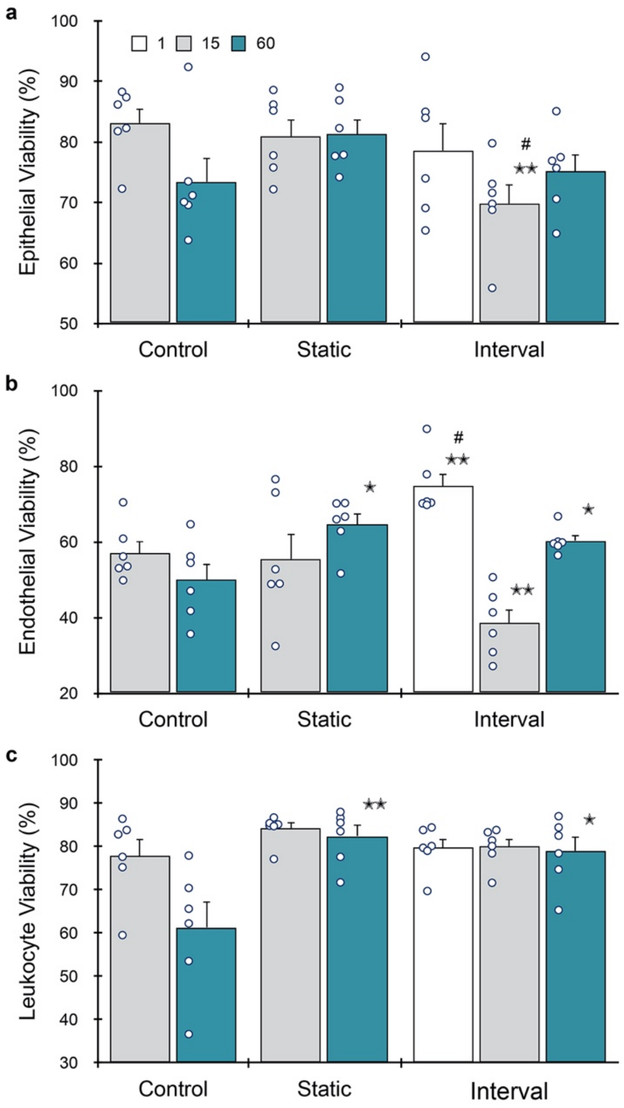

Supplementary Figure 11. Cell viability from final microfluidic platform studies using murine breast tumor (n=6 independent samples). (a) Epithelial cell viability was ~70-80% for all conditions. (b) Endothelial cell viability was generally low at ~50-60%. However, the 1 min device interval was higher at 75%, while the 60 min control and 15 min device interval were lower at 50% and 40%, respectively. (c) Leukocyte viability remained ~80% for all but the 60 min control, which was ~60%. Data are presented as mean values +/- SEM from at least three independent experiments. Circles indicate values for experimental replicates. Two-sided T test was used for statistical testing. Stars indicate p < 0.05 and double stars indicate p < 0.01 relative to the control at the same digestion time. Cross-hatches indicate p < 0.05 relative to the

static condition at the same digestion time. p-values for all comparisons are presented in the Source Data file.

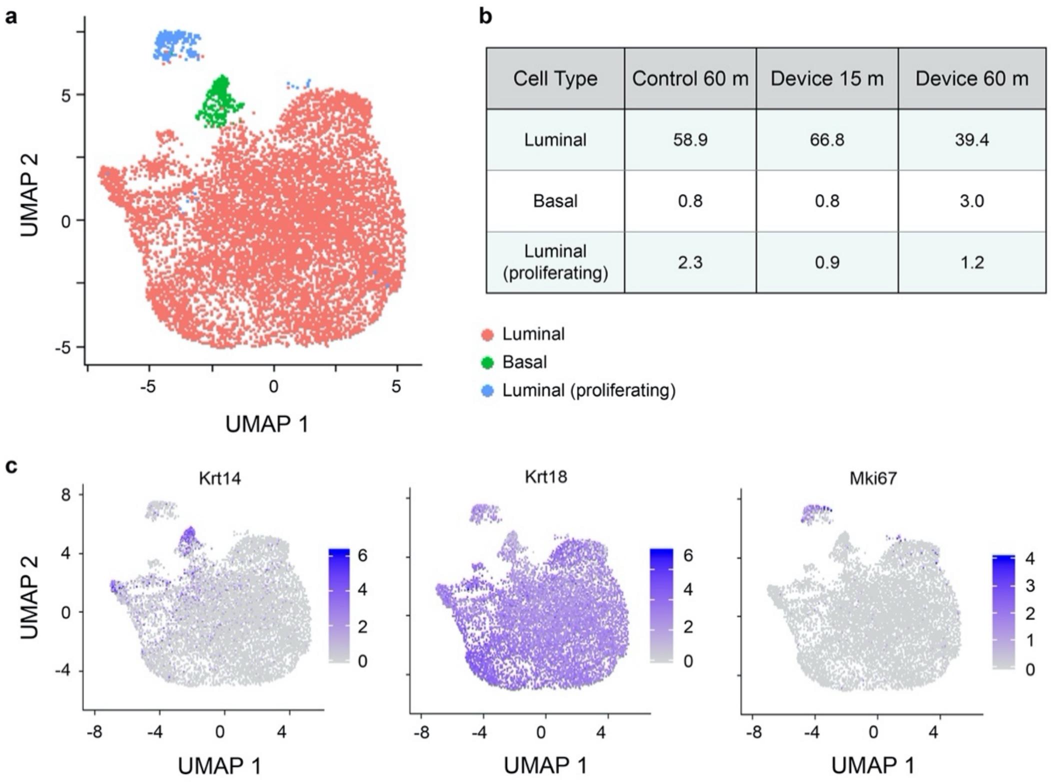

Cell Type	Control 60 m	Device 15 m	Device 60 m
Luminal	58.9	66.8	39.4
Basal	0.8	0.8	3.0
Luminal (proliferating)	2.3	0.9	1.2

Supplementary Figure 12. Sub-clustering epithelial cells for murine breast tumor. (a) The epithelial cluster contained 3 distinct sub-clusters that corresponded to luminal, basal, and proliferating luminal. (b) Population distributions in each sub-cluster. Luminal cells were enriched in the 15 min interval, while basal cells were enriched at 60 min. (c) The sub-clusters were identified primarily based on expression of *Krt14* (basal), *Krt18* (luminal), and *Mki67* (proliferating) genes.

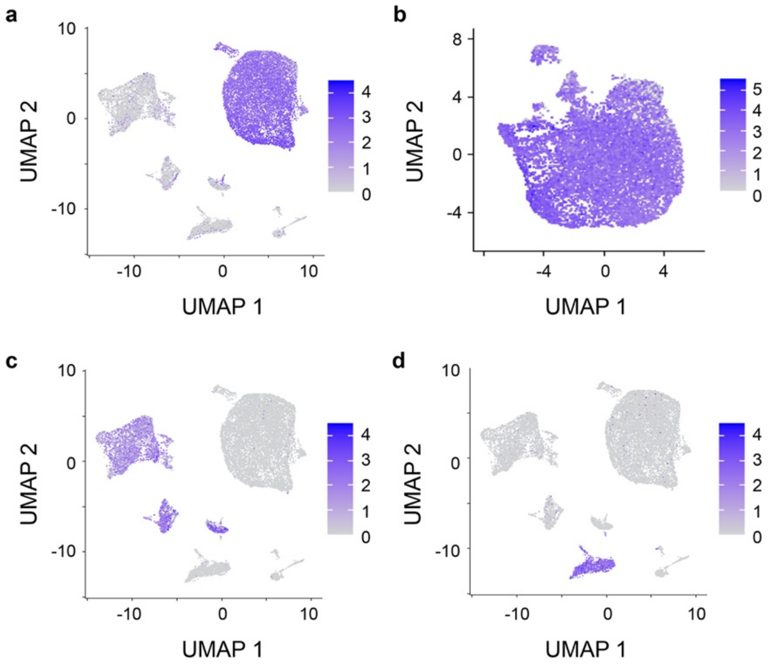

Supplementary Figure 13. Expression of *EpCAM*, *CD45*, and *CD31* in breast tumor clusters. (a,b) *EpCAM* was highly expressed within the (a) epithelial cluster and (b) each sub-cluster. (c) *CD45* was highly expressed in the macrophage, T lymphocyte, and granulocyte clusters. (d) *CD31* was highly expressed in the endothelial cluster.

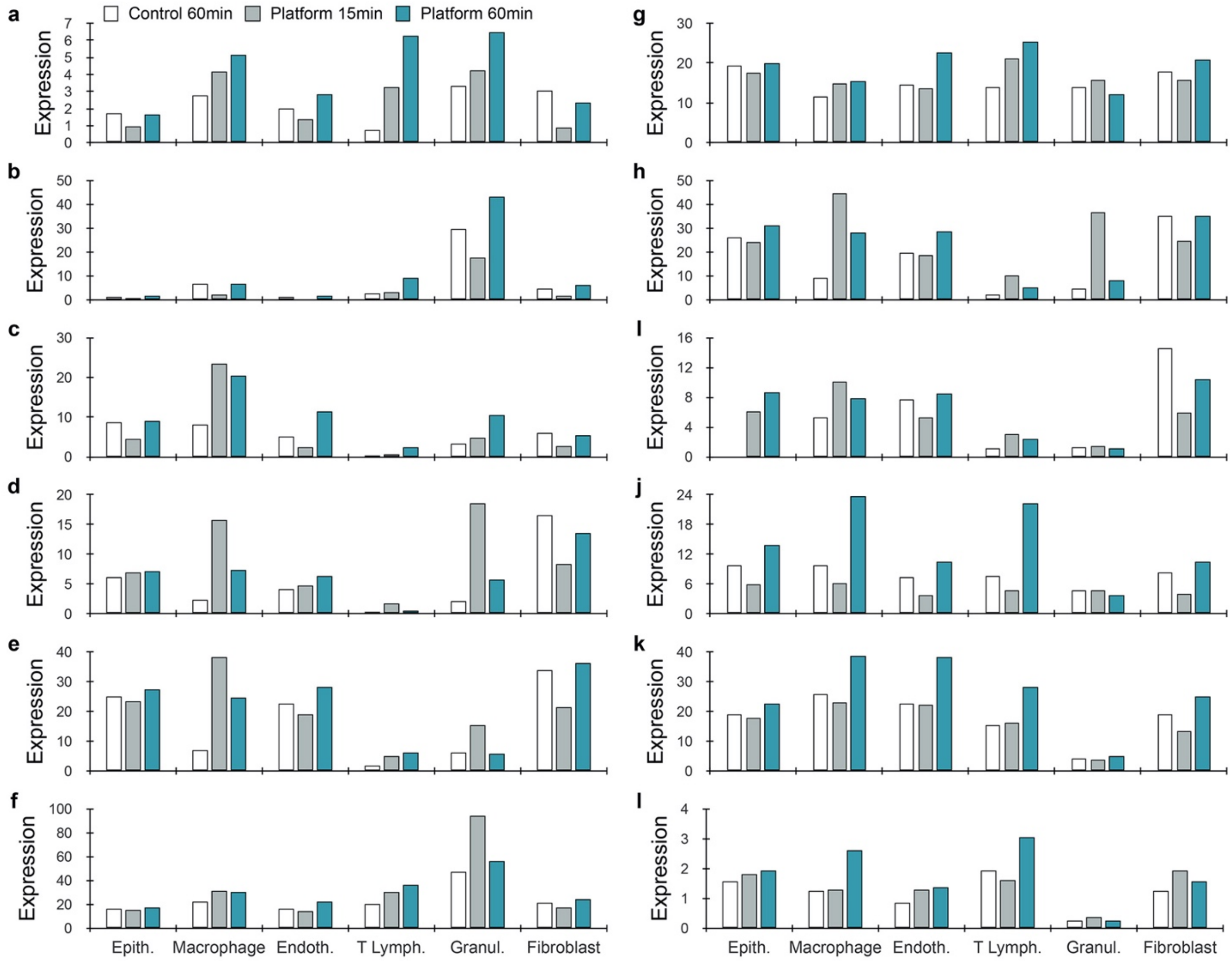

Supplementary Figure 14. Expression of select stress response genes for each breast tumor cell cluster. Average gene expression for common stress response genes including (a) *Nr4a1*, (b) *Gadd45b*, (c) *Atf3*, (d) *Egr1*, (e) *Jun*, (f) *Junb*, (g) *Jund*, (h) *Fos*, (i) *Fosb*, (j) *Hsp90aa1*, (k) *Hspa8*, and (l) *Hspd1*.

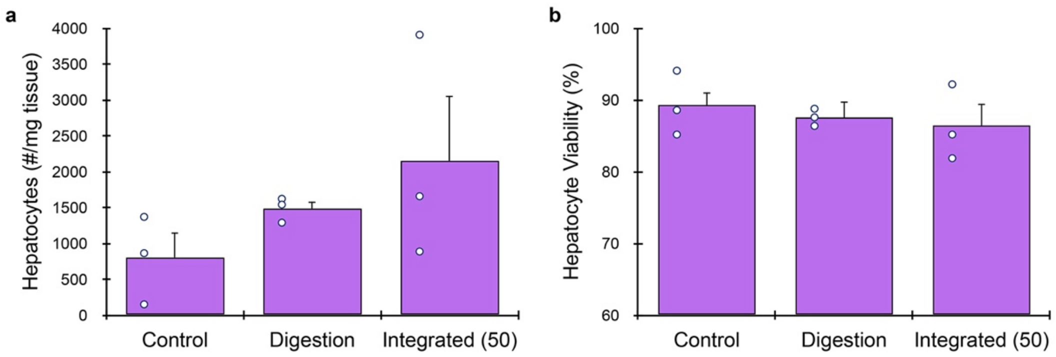

Supplementary Figure 15. Device optimization studies using murine liver (n=3 independent samples). Liver was processed with the minced digestion for 15 min and passed through the modified dissociation/filter device (50 μm filter only). (a) Hepatocytes increased by 30% relative to the digestion device alone and by nearly 3-fold relative to 15 min control. (b) Hepatocyte viability was >85% for all conditions. Data are presented as mean values +/- SEM from at least three independent experiments. Circles indicate values for experimental replicates. p-values for all comparisons are presented in the Source Data file.

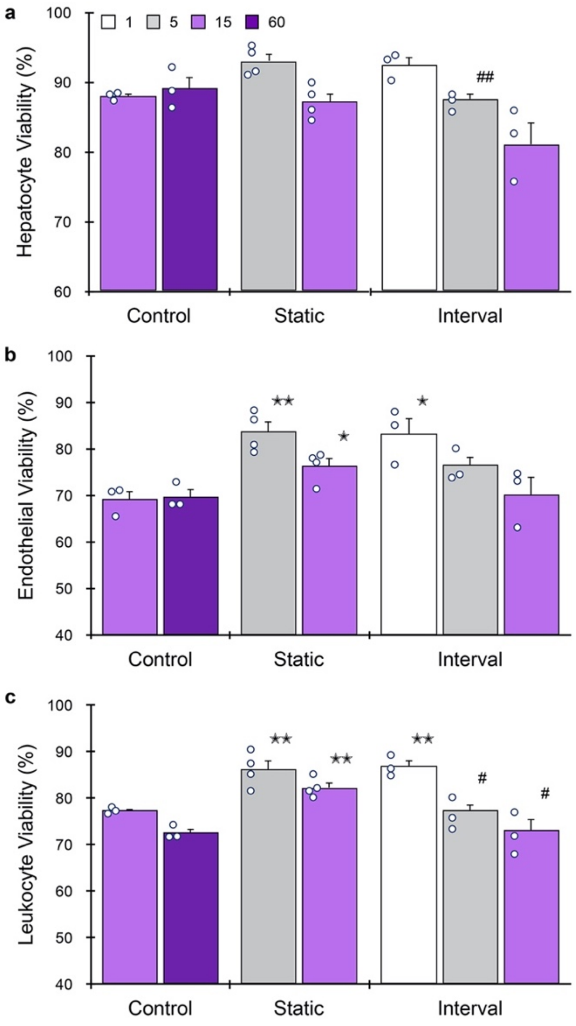

Supplementary Figure 16. Cell viability from final microfluidic platform studies using murine liver (n=3 or 4 independent samples). (a) Hepatocyte viability remained ~90% for all conditions except the 60 min interval, which decreased to ~85%. (b) Endothelial cell and (c) leukocyte viabilities were generally between ~70% and 85%, and increased with device processing at the early time points. Data are presented as mean values +/- SEM from at least three independent experiments. Circles indicate values for experimental replicates. Two-sided T test was used for statistical testing. Stars indicate $p < 0.05$ and double stars indicate $p < 0.01$ relative to the 60 min control. Cross-hatches indicates $p < 0.05$ and double cross-hatches indicate $p < 0.01$ relative to the static condition at the same digestion time. p-values for all comparisons are presented in the Source Data file.

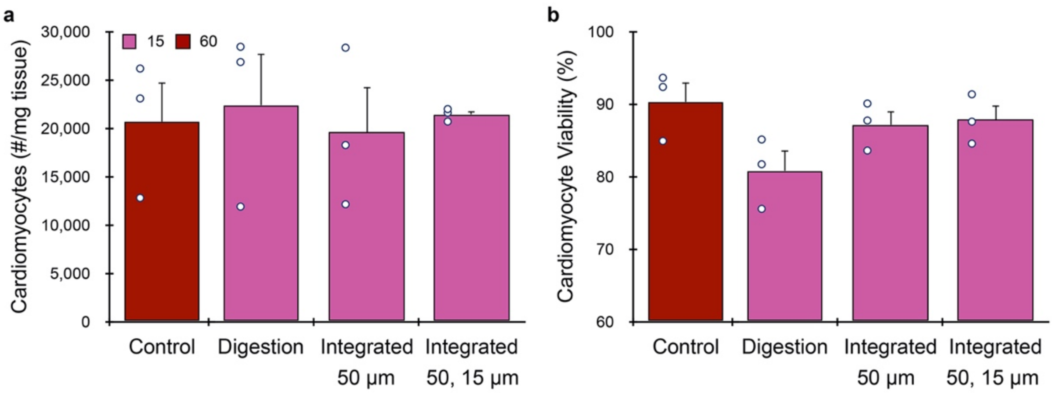

Supplementary Figure 17. Device optimization studies using murine heart (n=3 independent samples). Heart was processed with the minced digestion device for 15 min and passed through the integrated dissociation/filter with the original (50 and 15 µm filters) or modified (50 µm filter only) format. (a) Cardiomyocyte yield and (b) viability were similar for all conditions. Data are presented as mean values +/- SEM from at least three independent experiments. Circles indicate values for experimental replicates.

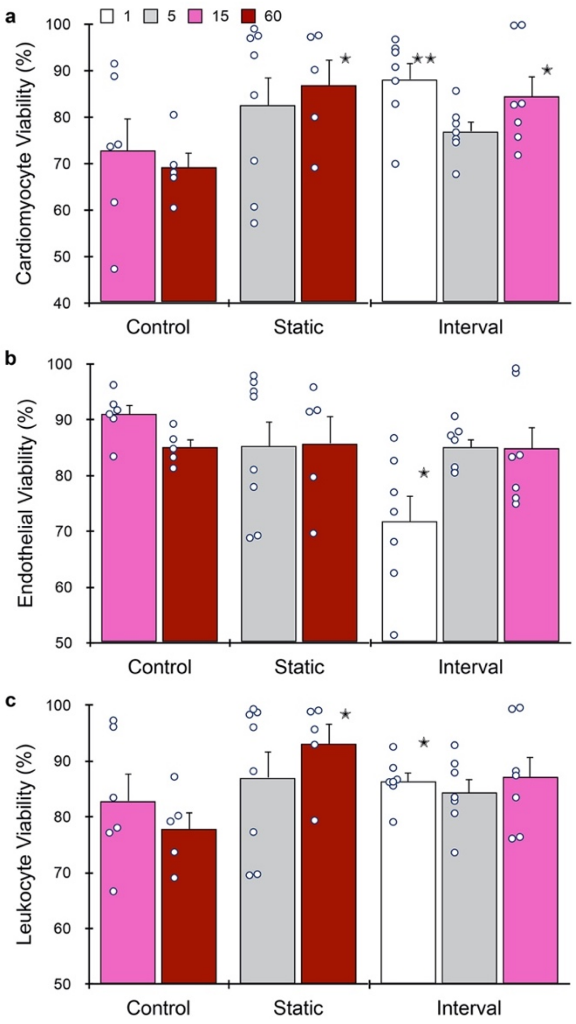

Supplementary Figure 18. Cell viability from final microfluidic platform studies using murine heart (n=5 to 8 independent samples). (a) Cardiomyocyte viability for device processed samples matched or exceeded controls. (b) Endothelial cell and (c) leukocyte viability was generally >80% for device and control conditions. Data are presented as mean values +/- SEM from at least three independent experiments. Circles indicate values for experimental replicates. Two-sided T test was used for statistical testing. Stars indicate $p < 0.05$ and double stars indicate $p < 0.01$ relative to the 60 min control. p-values for all comparisons are presented in the Source Data file.

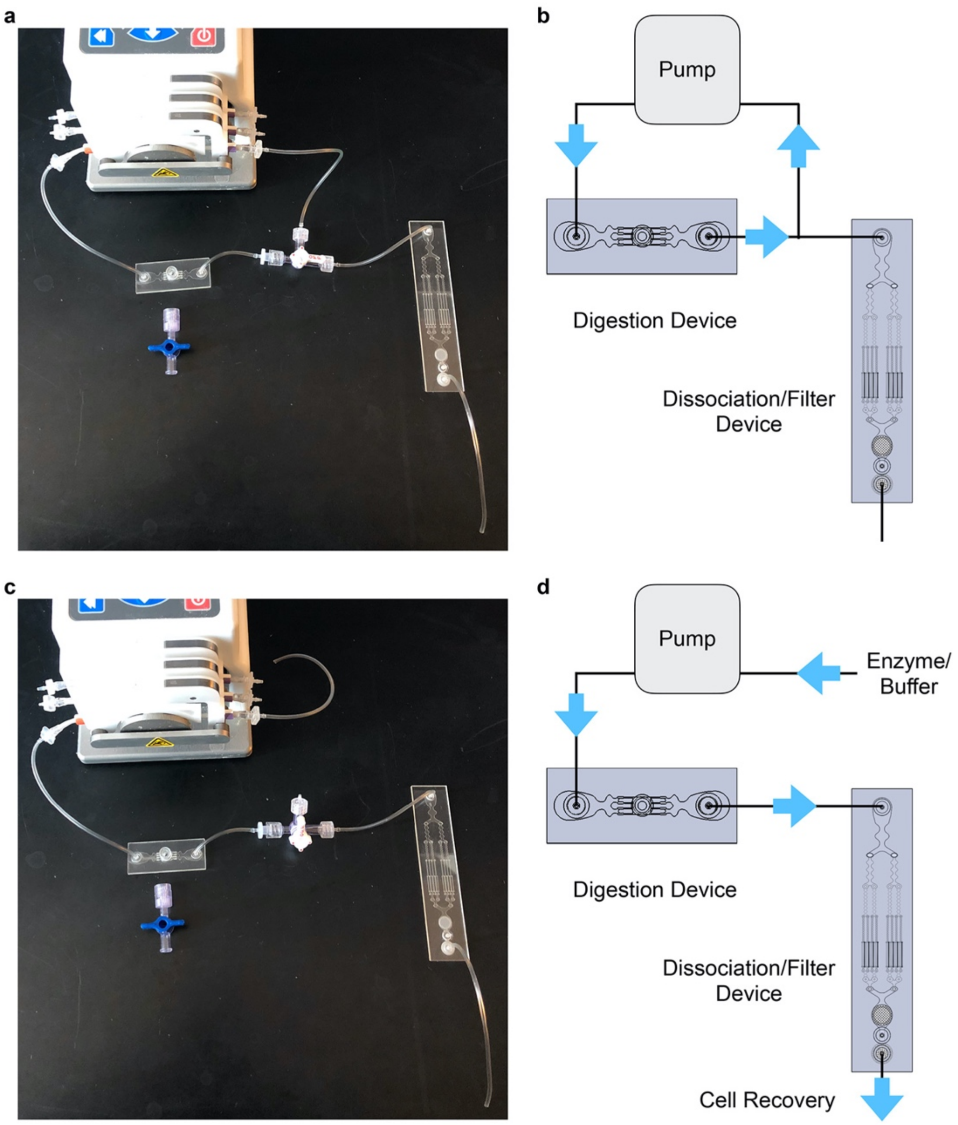

Supplementary Figure 19. Integrated fluidic system. (a,c) Picture of the system including peristaltic pump, digestion device, dissociation/filter device, and connections via valving and tubing. Including are configurations used for (a) recirculation through the digestion device and (c) sample elution during intervals or at the end of the run. (b,d) Schematics depicting flow paths, inputs, and outputs under (b) recirculation and (d) elution. For elution, fresh enzyme solution was used for intervals and buffer was used for final elution.

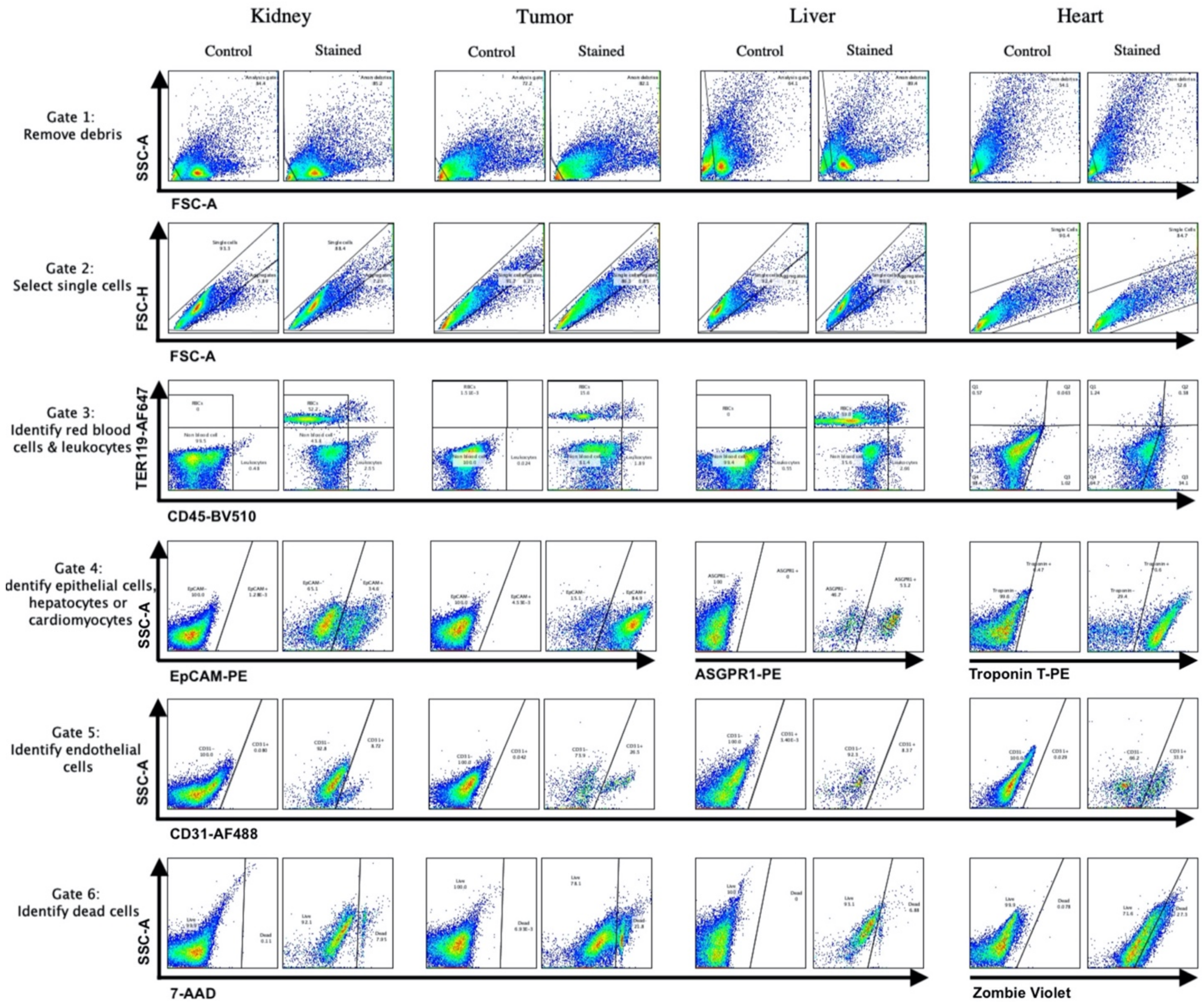

Supplementary Figure 20. Flow cytometry gating schemes. Cell suspensions were stained fluorescent probes (listed in Table 1) and signals were assessed by flow cytometry. Data was then analyzed using a sequential gating scheme. Gate 1 used FSC-A vs. SSC-A to exclude debris near the origin. Gate 2 used FSC-A vs. FSC-H to select single cells. Gate 3 used CD45-BV510 vs. TER119-AF647 to distinguish leukocytes (CD45+TER119-) and red blood cells (CD45-TER119+). Gate 4 was applied to the CD45-TER119- subset, and used PE to identify epithelial cells via EpCAM (kidney and tumor), hepatocytes via ASGPR1 (liver), or cardiomyocytes via Troponin T (heart). Gate 5 was applied to the EpCAM/ASGPR1/Troponin T negative cell subset and used CD31-AF488 to identify endothelial cells. Finally, gate 6 used 7-AAD (kidney, tumor, liver) or Zombie Violet (heart) to distinguish live and dead cells.

REFERENCES

1. Qiu, X. *et al.* Microfluidic channel optimization to improve hydrodynamic dissociation of cell aggregates and tissue. *Nat. Sci. Reports* 8, 2774 (2018). https://doi.org/10.1038/s41598-018-20931-y

2. Qiu, X., De Jesus, J., Pennell, M., Troiani, M. & Haun, J. B. Microfluidic device for mechanical dissociation of cancer cell aggregates into single cells. *Lab Chip* 15, 339-350 (2015). https://doi.org/10.1039/C4LC01126K

3. Qiu, X. *et al.* Microfluidic filter device with nylon mesh membranes efficiently dissociates cell aggregates and digested tissue into single cells. *Lab Chip* 18, 2776–2786 (2018). https://doi.org/10.1039/C8LC00507A

4. Tirosh, I. *et al.* Dissecting the multicellular ecosystem of metastatic melanoma by single-cell RNA-seq. *Science* 352, 189–196 (2016). https://doi.org/10.1126/science.aad0501

5. Park, J. *et al.* Single-cell transcriptomics of the mouse kidney reveals potential cellular targets of kidney disease. *Science* 360, 758–763 (2018). https://doi.org/10.1126/science.aar2131

6. Karaiskos, N. *et al.* A single-cell transcriptome atlas of the mouse glomerulus. *J. Am. Soc. Nephrol.* 29, 2060-2068 (2018). https://doi.org/10.1681/ASN.2018030238
