# Supplementary material for: Microfluidic platform accelerates tissue processing into single cells for molecular analysis and primary culture models
Source: Nat Commun. 2021 May 17;12:2858. doi: 10.1038/s41467-021-23238-1 (PMC8128882; doi:10.1038/s41467-021-23238-1)
Supplement: Supplementary file 3 — Reporting Summary [file 41467_2021_23238_MOESM3_ESM.pdf]

nature research

Corresponding author(s): Haun, Jered B

Last updated by author(s): Apr 13, 2021

Reporting Summary

Nature Research wishes to improve the reproducibility of the work that we publish. This form provides structure for consistency and transparency in reporting. For further information on Nature Research policies, see our Editorial Policies and the Editorial Policy Checklist.

Statistics

For all statistical analyses, confirm that the following items are present in the figure legend, table legend, main text, or Methods section.

n/a	Confirmed	
☐	☒	The exact sample size (n) for each experimental group/condition, given as a discrete number and unit of measurement
☐	☒	A statement on whether measurements were taken from distinct samples or whether the same sample was measured repeatedly
☐	☒	The statistical test(s) used AND whether they are one- or two-sided<br>*Only common tests should be described solely by name; describe more complex techniques in the Methods section.*
☒	☐	A description of all covariates tested
☒	☐	A description of any assumptions or corrections, such as tests of normality and adjustment for multiple comparisons
☐	☒	A full description of the statistical parameters including central tendency (e.g. means) or other basic estimates (e.g. regression coefficient) AND variation (e.g. standard deviation) or associated estimates of uncertainty (e.g. confidence intervals)
☐	☒	For null hypothesis testing, the test statistic (e.g. F, t, r) with confidence intervals, effect sizes, degrees of freedom and P value noted<br>*Give P values as exact values whenever suitable.*
☒	☐	For Bayesian analysis, information on the choice of priors and Markov chain Monte Carlo settings
☒	☐	For hierarchical and complex designs, identification of the appropriate level for tests and full reporting of outcomes
☒	☐	Estimates of effect sizes (e.g. Cohen's d, Pearson's r), indicating how they were calculated

Our web collection on statistics for biologists contains articles on many of the points above.

Software and code

Policy information about availability of computer code

Data collection	Flow cytometry: ACEA NovoExpress Software version 1.4.1. Next-generation sequencing: NovaSeq Control Software (NVCS) version 1.6.0 and Real-Time Analysis (RTA) version 3.4.4.
Data analysis	FlowJo version 10. 10x Genomics Cell Ranger software version 3.1.0.

For manuscripts utilizing custom algorithms or software that are central to the research but not yet described in published literature, software must be made available to editors and reviewers. We strongly encourage code deposition in a community repository (e.g. GitHub). See the Nature Research guidelines for submitting code & software for further information.

Data

Policy information about availability of data

All manuscripts must include a data availability statement. This statement should provide the following information, where applicable:

- Accession codes, unique identifiers, or web links for publicly available datasets
- A list of figures that have associated raw data
- A description of any restrictions on data availability

The authors declare that all data supporting the findings of this study are available within the article and its supplementary information files. The source data file was provided with this paper. All RNAseq data matrices along with their associated meta data have been deposited in the GEO database under accession code GSE163508 and SRA database under accession code PRJNA685210.

Field-specific reporting

Please select the one below that is the best fit for your research. If you are not sure, read the appropriate sections before making your selection.

- [x] Life sciences
- [ ] Behavioural & social sciences
- [ ] Ecological, evolutionary & environmental sciences

For a reference copy of the document with all sections, see nature.com/documents/nr-reporting-summary-flat.pdf

Life sciences study design

All studies must disclose on these points even when the disclosure is negative.

Sample size	Given that this study employed new devices and involved optimization, no sample size calculations were performed prior to experiments. Instead we included manually digested controls as a comparison, and for both devices and controls, tested different digestion times. For dissociation/filtering studies, different processing conditions were tested and compared to digestion alone.
Data exclusions	No data was excluded.
Replication	We focused on consistency between independent experimental replicates (different mice) for each tissue model. We performed at least 3 independent replicates (3 different mice) for all flow cytometry tests. If results from experimental replicates were reliable and differences with controls were statistically significant, sample size was deemed sufficient at 3. Otherwise, additional replicates were performed to obtain these metrics. A single replicate was used for single cell RNA sequencing due to cost associated with the method. All attempts at replications were successful and data is included in the results.
Randomization	Randomization was not required. All testing conditions for each replicate, including controls and device conditions, were performed on tissue from the same source mouse.
Blinding	Blinding was not required because the same tissue source mouse was used for all testing conditions.

Reporting for specific materials, systems and methods

We require information from authors about some types of materials, experimental systems and methods used in many studies. Here, indicate whether each material, system or method listed is relevant to your study. If you are not sure if a list item applies to your research, read the appropriate section before selecting a response.

Materials & experimental systems

n/a	Involved in the study	
[ ]	[x]	Antibodies
[ ]	[x]	Eukaryotic cell lines
[x]	[ ]	Palaeontology and archaeology
[ ]	[x]	Animals and other organisms
[x]	[ ]	Human research participants
[x]	[ ]	Clinical data
[x]	[ ]	Dual use research of concern

Methods

n/a	Involved in the study	
[x]	[ ]	ChIP-seq
[ ]	[x]	Flow cytometry
[x]	[ ]	MRI-based neuroimaging

Antibodies

Antibodies used	Antibody information and staining concentrations: 5 µg/mL anti-mouse CD45-AF488 (clone 30-F11, Cat. # 103122, BioLegend, San Diego, CA), 7 µg/mL EpCAM-PE (clone G8.8, Cat. # 118206, BioLegend, San Diego, CA), and 5 µg/mL TER119-AF647 (clone TER-119, Cat. # 116218 BioLegend, San Diego, CA), 12.5 µg/mL CD45-BV510 (Clone 30-F11, Cat. # 103138, BioLegend, San Diego, CA), 8 µg/mL CD31-AF488 (clone MEC13.3, Cat. # 102514, BioLegend, San Diego, CA), 10 µg/mL ASGPR1-PE (clone 8D7, Cat. # sc-52623 PE, Santa Cruz Biotechnology, Dallas, TX), 0.15 µg/mL Troponin T (clone REA400, Cat. # 130-120-545, Milentyi Biotec, San Diego, CA).
Validation	Each antibody was tested using the appropriate tissue type and, if possible, cell lines. Antibodies were validated by the manufacturer for the application of flow cytometry and for murine species.

Eukaryotic cell lines

Policy information about cell lines

Cell line source(s)	MCF7 cells were obtained from ATCC
Authentication	None of the cell lines were authenticated

Mycoplasma contamination	Cell lines tested negative for mycoplasma
Commonly misidentified lines (See ICLAC register)	No commonly misidentified lines were used in this study.

Animals and other organisms

Policy information about studies involving animals; ARRIVE guidelines recommended for reporting animal research

Laboratory animals	BALB/c or C57B/6 mice were used for normal kidney, liver, and heart experiments. MMTV-PyMT were used for tumor studies. We used both male and female mice, and ages were approximately 6 weeks. All mice were maintained at the University of California, Irvine according to the guidelines of the Institutional Animal Care and Use Committee.
Wild animals	No wild animals were used in the study.
Field-collected samples	No field collected samples were used in this study.
Ethics oversight	BALB/c or C57B/6 mice were deemed waste from IACUC approved studies at UCI. An IACUC protocol was approved for MMTV-PyMT mouse studies.

Note that full information on the approval of the study protocol must also be provided in the manuscript.

Flow Cytometry

Plots

Confirm that:

- [x] The axis labels state the marker and fluorochrome used (e.g. CD4-FITC).
- [x] The axis scales are clearly visible. Include numbers along axes only for bottom left plot of group (a 'group' is an analysis of identical markers).
- [x] All plots are contour plots with outliers or pseudocolor plots.
- [x] A numerical value for number of cells or percentage (with statistics) is provided.

Methodology

Sample preparation	Protocols are described in detail in the methods section. This study used cells resulting from 4 tissues, and sought to quantify the numbers of endothelial, leukocyte, and red blood cells from each tissue. Additional markers were used in a tissue-specific manner (EpCAM for kidney and tumor, ASGPR1 for liver/hepatocytes, traponin T for heart/cardiomyocytes)
Instrument	Novocyte 3000 Flow Cytometer (ACEA Biosciences) was used for analysis of cell populations. A FACSAria Fusion (BD Biosciences) was used for cell sorting prior to single cell RNA sequencing.
Software	Data was analyzed using FlowJo
Cell population abundance	Cell population abundance was reported as bar graphs of percent total for each processing condition for each tissue type.
Gating strategy	The gating strategy is clearly outlined in the supplementary information methods. A supplementary figure is also included to show representative samples.

- [x] Tick this box to confirm that a figure exemplifying the gating strategy is provided in the Supplementary Information.
